# Lecturers’ perceptions on student midwives’ competence in symphysis fundal height measurement

**DOI:** 10.4102/hsag.v30i0.3054

**Published:** 2025-10-20

**Authors:** Winile D. Cele, Pretty N. Mbeje, Euphemia M. Mhlongo

**Affiliations:** 1 School of Nursing and Public Health, College of Health Sciences, University of KwaZulu-Natal, Durban, South Africa

**Keywords:** lecturers, perceptions, student midwives, competence, confidence, symphysis fundal height

## Abstract

**Background:**

Performing midwifery care requires competence and confidence because of the profession’s emphasis on independent functioning and professional responsibility. The student midwives generally can manage to plot the symphysis fundal height measurements on charts correctly, but their real learning need is to accurately interpret what the measurements meant in relation to their patients’ due dates and overall pregnancy health. This interpretation is essential for the early detection of potential complications.

**Aim:**

To explore and describe lecturers’ perceptions of student midwives’ competence and confidence in performing symphysis fundal height measurements, plotting, and interpreting the results.

**Setting:**

The study took place in KwaZulu-Natal College of Nursing, South Africa.

**Methods:**

A descriptive, qualitative design was used. Data were collected using focus group discussions from August 2023 to September 2023. Thematic analysis method was used to analyse data.

**Results:**

Four main themes emerged namely, perceived significance of symphysis fundal height measurement, the perceived prerequisites enabling competence and confidence, the perceived challenges and barriers to competence and confidence, and the recommendations made to improve the competence and confidence of student midwives.

**Conclusion:**

It was the perception of the lecturers that improved competence and confidence of student midwives in the measurement, plotting, and interpretation of symphysis fundal height could lead to the correct interpretation and identification of high-risk pregnancies.

**Contribution:**

The findings will guide the curriculum developers and policy makers in the formulation of symphysis fundal height measurement protocols and standard operating procedures, to improve and standardise midwifery skills in this regard.

## Introduction

The International Confederation of Midwives (ICM) (2024) aims at strengthening midwifery skills globally. The ICM outlines the essential competences for midwifery practice and states that a midwife should possess the requisite knowledge and skills when she is practising. The ICM competences comprise five categories (ICM 2024). In this study, the researchers focused on category three, which describes the antenatal care of a woman. This category deals with assessing the health of both mother and foetus, promoting their health and well-being, and detecting risks during pregnancy. The symphysis fundal height (SFH) measurement is one of the antenatal measurements taken and a skill that is performed by a midwife to monitor foetal growth.

This study is part of the main mixed-method study conducted to develop the guidelines for SFH measurement for the student midwives. The developed guidelines will not replace the existing South African maternity care guidelines that are currently being used in the clinical setting (Government 2024). As part of the main study, a scoping review of literature was conducted to map the methods used to estimate the gestational age during pregnancy in the African continent.

### Background

The SFH is measured from the superior border of the symphysis pubis to the highest point of the uterine fundus (Chen et al. 2020, Sellers 2018). It is the basic antenatal tool that is used to determine the gestational age during pregnancy and to exclude multiple pregnancies, polyhydramnios and intra-uterine growth retardation (IUGR). Symphysis fundal height measurement is also used to identify large for gestational age (Goto 2020).

Globally, the SFH measurement is used to monitor foetal growth. In low- and middle-socio-economic countries, the SFH measurement is used to estimate foetal growth and the gestational age in the absence of ultrasound and menstrual data (Basso et al. 2020; Cronje, Cillierrs & Du Toit 2016; González-Fernández et al. 2021; Lee et al. 2020). The SFH measurement is used internationally, but there was a previous lack of standardisation of the process because while some countries used the international SFH calculator, which is an Excel-based application, to produce the required centiles and *z*-scores, others used SFH graphs and charts to produce the centiles and *z*-scores. In view of this lack of standardisation, Papageorghiou et al. (2017) developed a new international SFH standard using data from eight countries, namely Brazil, China, India, Italy, Kenya, Oman, the United Kingdom and the United States. González-Fernández et al. (2021) subsequently conducted a study where this new standard was used to assess the SFH and reported that few studies had applied and assessed the new SFH standard at that time.

Basso et al. (2020) reported that the SFH measurement is an acceptable method to estimate the gestational age. They also compared the SFH curve for diabetic and non-diabetic women and determined that SFH is also useful in the prenatal care of women with gestational diabetes mellitus (Basso et al. 2020). However, Valderrama et al. (2020) opined that the method cannot be used alone and must be used together with the last menstruation date to obtain more accurate results. This is in line with the findings of Self et al. (2022) where they used different methods such as last normal menstrual period (LNMP) confirmed by ultrasound (crown rump length [CRL]) because some of the women are not sure of their dates, SFH measurements and *in vitro* fertilisation dates to estimate the gestational age. Crown rump length measured before 14 weeks is regarded as a gold standard for gestational ageing (Self et al. 2022; Whelan et al. 2022). Crown rump length measured in the second and third trimesters is considered more accurate when more than one parameter is used (Self et al. 2022). Caradeux et al. (2024) concurred that the third-trimester ultrasound improves the detection rate of IUGR compared to SFH, although there is insufficient evidence. Intra-uterine growth retardation is a high-risk condition that contributes to foetal and neonatal mortality and morbidity and can be identified by SFH in low-risk pregnancy (Giouleka et al. 2023). In a high-risk pregnancy, IUGR can be detected by ultrasound, and for further management, Doppler ultrasound can be performed.

Other studies were conducted on pregnant mothers using the SFH measurement, LNMP and the abdominal circumference to estimate the gestational age. Lee et al. (2020) reported that although these parameters are acceptable for predicting the gestational age, they should be used together with ultrasound scans to obtain more accurate results. They thus recommended the use of ultrasonography and an increase in the number of trained ultrasonographers for this purpose. Wanyonyi and Mutiso (2018), however, highlighted the lack of adequately trained obstetric ultrasonographers, as they found that those who were performing ultrasound scanning had not undergone formal ultrasound training. To overcome the shortage of ultrasonographers, Uganda and Tanzania’s healthcare workers, including midwives, attended a face-to-face short course on ultrasound technology and scanning, while Mozambique’s healthcare workers received their training remotely through distance learning (Wanyonyi & Mutiso 2018).

Fung et al. (2020) recommended that ultrasound scanning be carried out before 24 weeks of gestation when estimating the gestational age. In sub-Saharan countries, abnormal foetal growth is still a concern among healthcare workers. The reason for this is that it increases neonatal mortality and poses a risk of stillbirth. Wanyonyi and Mutiso (2018) reported on three methods that are currently used to monitor foetal growth in clinical practice, namely abdominal palpation, SFH measurements and ultrasound scans. Their findings revealed that there is poor access to ultrasound equipment in most areas with poor resources, even though it is regarded as the most reliable method for assessing foetal growth. Symphysis fundal height measurements and abdominal palpations are the two methods available in these low-resource settings to monitor foetal growth, and Wanyonyi and Mutiso (2018) consider them unreliable.

Ariyo, Yohanna and Jo (2020) conducted a quantitative study in Nigeria, which aimed to determine the accuracy of the SFH and abdominal girth measurements in estimating foetal birth weights among expecting women. Their findings showed a significant correlation between the estimated foetal weights and the actual birth weights; 66% of normal weight babies and 87.5% of macrosomic babies were predicted correctly (Ariyo et al. 2020). Their findings contradicted those of Wanyonyi and Mutiso (2018), who considered SFH and abdominal palpation measurements unreliable.

The South African maternal and neonatal health policy (Government 2021) emphasises the importance of effective referral pathways to facilitate access to the higher level of care required for further investigation and management of at-risk pregnancies. Prompt and appropriate responses to these pregnant mothers’ needs can improve their pregnancy outcomes. One of the factors that hamper the referral system is the shortage of resources, including sufficient staff with adequate obstetric skills, as indicated in the Saving Mothers’ report of 2019–2022 (Government 2022). Correct SFH measurement is one of the obstetric skills that can improve maternal health outcomes in South Africa, because the SFH measurement is used to estimate gestational age and to monitor foetal growth.

The SFH measurement is a basic tool used by the midwives in the study area of KwaZulu-Natal. However, during clinical accompaniment of students a gap was identified: the SFH measurements were not plotted in some of the maternity records in some of the hospitals in the study area. This was also identified by Dlamini (2023), who conducted a study to monitor foetal growth in KwaZulu-Natal and reported that in some of the maternity case records, the SFH measurements were either recorded incompletely or not recorded at all. Some of the reasons provided for not recording the SFH results correctly were incompetence when taking the measurements and plotting them and shortages of staff. The researchers therefore decided to conduct this study to explore and describe the perceptions of nursing lecturers on the competence and confidence of student midwives in measuring the SFHs, plotting the results on the graphs and interpreting the results. The findings of this study will assist the students in performing the task correctly, thus improving their confidence in performing the task and helping prepare them for registration as competent midwives with the South African Nursing Council (SANC).

The researchers reviewed literature on the association between teaching strategies and self-confidence in clinical skills (Sharma, Hildingsson & Christensson 2019; Stone, Cooke & Mitchell 2020). The study by Stone et al. (2020) aimed to explore students’ experiences in using three clinical skills video podcasts and their confidence in practising the skills learned, whereas Sharma et al. (2019) conducted a study on teaching and learning methods and the self-confidence of student midwives. Sharma et al. (2019) reported that classroom teaching was the most practised method of teaching, followed by practical laboratory demonstrations, practising on models, demonstrations at clinical sites and attending births (the least practised). Basic midwifery students showed high levels of confidence in laboratory skills and supervised clinical skills during clinical placement. Furthermore, the nursing diploma students were more confident than the bachelor’s degree students. Stone et al. (2020) reported that undergraduate nursing students supported the use of video podcasts in learning clinical skills; however, the students still supported face-to-face contact. Stone et al. (2020) thus concluded that classroom teaching or face-to-face contact with the students should be used together with any other teaching method to reinforce learning and to broaden the students’ depth of understanding.

Further studies were conducted among bachelor’s degree and diploma student midwives to ascertain their level of confidence in antepartum, intrapartum, postpartum and newborn care (Bäck & Karlström 2020; Sharma et al. 2018). The diploma students showed higher confidence levels than the bachelor’s degree students, despite both groups having little clinical exposure (Sharma et al. 2018). Bäck and Karlström (2020) outlined the factors that affect student confidence, which are uninterested supervisors, patronising attitudes towards students, poor supervision during clinical exposure and poor relationships with the midwives during clinical exposure.

In South Africa, a study was conducted in the Western Cape to investigate self-perceived preparedness for midwifery students after completion of the final examination (Ramahlo & Mayers 2025). The findings revealed that the students had difficulty in performing basic skills. Low confidence level in managing women independently during breech delivery, twin delivery and breast examination was reported. Students lacked confidence, and they perceived themselves as unprepared to practise as registered midwives after completion of training (Ramahlo & Mayers 2025).

The researchers conducted this study to explore and describe the lecturers’ perceptions of student midwives’ competence and confidence in the measurement of the SFH, as no similar studies had been done in the South African context.

### Research aim

This study aimed to explore and describe the nursing lecturers’ perceptions of student midwives’ competence and confidence in performing SFH measurements, plotting the results onto graphs and interpreting the results at the selected campuses of the KwaZulu-Natal College of Nursing (KZNCN).

### Research

The objective of this study is to explore and describe the lecturers’ perceptions of student midwives’ competence and confidence in taking SFH measurements and plotting and interpreting the results.

## Research methods and design

### Research paradigm and design

Pragmatism was adopted because it is a paradigm that combines both qualitative and quantitative techniques rather than focusing on only one design. Pragmatism allows the researchers to choose the method that meets the purpose of the study. The qualitative technique involves subjectivity, reflexivity and contextual understanding. It focuses on in-depth insight into the lives of research participants and also aims to understand their behaviour (Creswell & Creswell 2018). Qualitative research design allows close

interaction with participants (Terre Blanche, Durkeim & Painter 2018). Hence, the researchers conducted focus group discussions (FGDs) to obtain in-depth information from the lecturers regarding the competence of student midwives in SFH measurement.

The study adopted an exploratory, descriptive, qualitative design to get new information and a better understanding of the phenomenon under study (McCombes 2022).

### Study setting

The study was conducted at three KZNCN campuses in KwaZulu-Natal province, South Africa. The KZNCN is physically located in Pietermaritzburg. It is a public nursing college accredited by the SANC and the Council of Higher Education (CHE), and it is registered with the South African Qualifications Authority (SAQA). The KZNCN has 11 campuses offering different nursing programmes. At the time of data collection, two undergraduate programmes were offered: R425, a 4-year diploma leading to registration as a General Nurse (Psychiatric and Community) and Midwife, which was being phased out, and R171, a 3-year diploma in nursing.

### Population and sampling

#### Population

Lecturers from the KZNCN campuses with midwifery experience, a nursing education qualification and a minimum of 3 years of experience in the midwifery department met the criteria for inclusion in the study. Lecturers with less than 3 years of experience in the midwifery department were excluded. In this study, lecturers mean nurse educators appointed by KZNCN who are involved in the teaching of the student midwives, namely midwifery lecturers, Head of Department – Midwifery and midwifery clinical lecturers.

#### Sample and sampling

A purposive sampling technique was used to select the eligible participants for the FGDs. Creswell and Creswell (2018) stated that in qualitative research, the people or sites that understand the central phenomenon under study are selected. The researchers obtained permission from the campus principals, who then communicated with the research committees to arrange suitable dates for the FGDs. The size of the sample was 15 participants, who participated in three focus groups. There were six participants from Campus A, four from Campus B and five from Campus C. According to Fouché, Strydom and Roestenburg (2022):

[*A*] focus group is a qualitative data collection method, where a heterogeneous or homogenous group with three to fifteen participants meets and engages in a focus group discussion on a proposed topic to generate diverse qualitative data.

#### Data collection

Data were collected from August 2023 to September 2023. The FGDs were conducted at the respective campuses. Prior arrangements were made with the campus principals to ensure the availability of the participants on the days of the FGDs. The FGD meetings lasted for 50 min to 60 min. All participants were given an information sheet that assisted the researcher in explaining the details of the research, and research ethics principles were discussed. The participants were asked to sign an informed consent form before the group discussions started. The researcher requested permission from the participants to record the FGDs and to take field notes as a backup method. The researcher used a cell phone with a password to record the discussions. All FGDs were conducted in well-ventilated rooms and all COVID-19 protocols were observed. The same interview schedule with nine questions outlined in Online Appendix 1 guided all three FGDs. The questions were formulated based on the findings of the quantitative phase of the main study (a mixed-method study).

#### Data analysis

The data obtained from the FGDs were analysed using the steps of thematic analysis. Braun and Clarke (2006) defined thematic analysis as ‘a qualitative data analysis method that involves reading through a data set (such as transcripts from in-depth interviews or focus groups) and identifying patterns in meaning across the data’. The researcher chose thematic analysis because it is flexible and it comprises strict descriptive data (Naeem et al. 2023).

The first step in qualitative analysis involves reading and re-reading to become familiar with the data. In this study, the data were in the form of audio files. The researcher transcribed them personally and read through the transcripts to identify meanings and patterns across the data (Naeem et al. 2023). The researcher made notes and wrote some points.

In the second step, an independent coder was used to organise and identify the initial codes that represented the meanings and patterns seen in the data. Data with the same meaning had the same code. The coding was determined by the research questions (Online Appendix 1).

In the third step, the themes were identified. The transcripts were compared to the field notes, and the context of each statement was checked. All identified codes were combined to form themes. Some codes were organised into main themes and subthemes.

In the fourth step, the themes identified in the third step were reviewed, and the data were categorised and confirmed to be meaningful (Braun & Clarke, 2006).

#### Rigour

The researchers ensured the quality of the evidence by using Shenton’s *trustworthiness* principles (Moser & Korstjens 2018; Shenton 2004). The lecturers who participated in the FGDs were selected using a purposive sampling technique, although Shenton (2004) prefers a random sampling of participants. During data collection, the researcher used probing questions according to the interview guide, as advised by Shenton (2004) and Moser and Korstjens (2018). After data collection, the researcher transcribed the recordings. *Transferability* was ensured by reviewing the transcripts and listening repeatedly to the recordings (Shenton 2004). The transcripts were coded by the independent coder, and the findings were summarised. After data analysis, the researcher reviewed the findings with her peers, followed by debriefing sessions with the research supervisor for verification of the data. The researcher verified the perceptions of the lecturers about the student midwives’ competence and confidence in SFH measurement, plotting and interpretation to ensure the authenticity of the results (Moser & Korstjens 2018). The findings were also verified to see if they were consistent with the raw data that were collected to ensure *confirmability*. Confirmability was also ensured by using extracts of the participants’ quotes to support the subthemes identified. The researcher used the same interview guide during the three FGDs to ensure consistency of the findings. This is supported by Lincoln and Guba (as cited by Moser & Korstjens 2018; Shenton 2004), who mentioned that *dependability* includes the aspect of consistency.

### Ethical considerations

Ethical approval was obtained from the University of KwaZulu-Natal’s (UKZN) Research Committee (BREC/00004960/2022) and the Provincial Department of Health (DOH) Ethics Committee (KZ_202302_027). Gatekeeper permission was also obtained from KwaZulu-Natal College of Nursing (KZNCN). All participants were given information sheets to ensure that they understood the purpose of the study. The participants were informed that their participation was voluntary and that they could withdraw from the study at any time. They all consented to participate in the study and to be audiotaped during the focus group discussions (FGDs). The FGDs were conducted in private rooms to maintain confidentiality. The audio recordings and transcripts were stored in a computer with a password known only to the researcher. Numbers were used instead of participants’ names during the FGDs and during the reporting of the findings.

## Results

### Demographic characteristics

The demographic data of the participants showed their occupational rank and experience in the midwifery field. The demographic characteristics are displayed in Table 1.

**TABLE 1:** Participants’ demographic data (*N* = 15).

Participant	Occupation	Years of service as a registered midwife	Years of service in the midwifery department
**Campus A (FGD1)**			
Participant 1	Lecturer	7	7
Participant 2	Clinical lecturer	32	21
Participant 3	Lecturer	8	7
Participant 4	Lecturer	20	10
Participant 5	Lecturer	30	20
Participant 6	Lecturer	30	27
**Campus B (FGD2)**			
Participant 7	Lecturer	29	15
Participant 8	Clinical lecturer	32	20
Participant 9	HOD-Midwifery	20	20
Participant 10	Lecturer	33	30
**Campus C (FGD3)**			
Participant 11	HOD-Midwifery	32	32
Participant 12	Lecturer	34	34
Participant 13	Lecturer	33	3
Participant 14	Clinical lecturer	13	4
Participant 15	Lecturer	35	9

Note: Participant 1 (P1), Campus A (FGD 1), Campus B (FGD2), Campus C (FGD3).

FGD, focus group discussion.

The FGD participants comprised two Midwifery Heads of Department, 10 lecturers and three clinical lecturers. They all met the inclusion criteria of being lecturers with a nursing education qualification and a minimum of 3 years full-time experience in the midwifery department. From the three campuses that participated in this study, there were 12 lecturers with 20–35 years full-time experience as registered midwives, and 8 lecturers with 20 years and above of experience in the midwifery department. In the excerpts below, the participants are listed as P1 (participant 1) to P15. FGD1 refers to the FGD held on Campus A, FGD2 refers to the one held on Campus B and FGD3 refers to the one held on Campus C.

### Focus group discussion questions

The three FGDs were guided by the following questions outlined in Online Appendix 1:

- From your experience as a midwife, would you consider it significant to measure the SFH at every antenatal visit? Please support your statement.
- What do you think should be the prerequisites for SFH measurement?
- As a lecturer, which teaching strategies do you think are suitable for teaching the SFH measurement?
- How do you ensure that the SFH graph is plotted correctly on the SFH chart during the teaching session?
- During the clinical accompaniment of the students, did you find all the SFH graphs plotted on the SFH charts?
  ▪ If not, what do you think caused that to occur?
- How would you ensure that the SFH graph and observations on the SFH chart are recorded accurately?
  ▪ What are the implications if the above recordings are inaccurate?
- As a lecturer, which method do you think is suitable for assessing the competence of the student midwives in SFH measurement, plotting and interpretation?
- During clinical assessment, what was the level of competence and confidence in SFH measurement, plotting and interpretation?
- As a midwife, what do you think should be included in the development of the SFH guidelines for the student midwives?

### Themes and subthemes

The four themes that emerged during the data analysis are displayed in Table 2. They are: (1) perceived significance of the SFH measurement, plotting and interpretation; (2) perceived prerequisites enabling competence and confidence; (3) perceived challenges and barriers to competence and confidence; and (4) recommendations to improve the competence and confidence of students in SFH measurement.

**TABLE 2:** Themes and subthemes.

Themes	Subthemes
1. Perceived significance of the SFH measurement, plotting, and interpretation of the results.	1.1. Cost-effective, non-invasive monitoring tool. 1.2. Fosters mothers’ wellbeing. 1.3. Early identification of complications and initialisation of interventions.
2. Perceived prerequisites enabling competence and confidence in performing the SFH measurement.	2.1. Prior theoretical knowledge of the SFH procedure. 2.2. Correct interpretation and documentation of the SFH results. 2.3. An enabling therapeutic environment. 2.4. Utilising the correct clinical teaching strategies.
3. Perceived challenges and barriers to competence and confidence in performing the SFH procedure.	3.1. Training was done in different institutions. 3.2. Limited resources for students’ SFH training. 3.3. Negative attitude. of students towards the SFH procedure. 3.4. Limited prior knowledge, which affected their confidence and competence.
4. Recommendations to improve competence and confidence when taking SFH measurements and plotting and interpreting the results.	4.1. Standardised practice. 4.2. Availability of resources and training of educators. 4.3. Multi-disciplinary team collaboration.

SFH, symphysis fundal height.

#### Theme 1: Significance of symphysis fundal height measurement, plotting and interpretation

The participants indicated perceived significance of SFH measurement, plotting and interpretation to be cost-effective, accurate and non-invasive monitoring tool, encouraging the mother’s well-being and enhancing early identification of complications and initialisation of interventions.

**Cost-effective, accurate and non-invasive monitoring tool:** Participants indicated that SFH is cost-effective and is an accurate tool readily available for midwives at every level of care, including areas where there are no ultrasound scans. Although SFH does not give any clinical value in terms of how the foetus is growing before 20 weeks and after 36 weeks, it is a very important measure as it gives you an indication of the height of the fundus from 20 to 36 weeks. As a tool, SFH measurement was preferred for its ease of use at every level of care from PHC clinics to high-level hospitals. Participants also expressed that SFH measurement gives an opportunity to observe the well-being of the mothers:

‘It is a non-invasive tool, it is fast to diagnose, it can be used at any level of care and knowing the SA health care system we might not have any other way of assessing normality or the abnormality of the growth of the foetus and I think about things like the ultrasound is not always available but SFH is always available.’ (P4, FGD1)

‘It is very important that the patient must be 20 weeks and over to really do a fundal height properly. And also, to compare with the estimated date of delivery [*EDD*].’ (P11, FGD3)

**Enhances early identification of complications and initialisation of interventions:** Early identification of the abnormal growth of the uterus assists in the identification of high-risk pregnancy, namely multiple pregnancies, pre-eclampsia, polyhydramnios or oligohydramnios. Any deviation from normal requires prompt management, thus potentially saving the life of both mother and the foetus. Moreover, SFH can be used as a form of triaging pregnant mothers. It also gives an indication of the height of the fundus and whether it is corresponding to the dates. Therefore, early diagnosis and management are advantageous in saving the life of the mother and the baby:

‘I also feel that it is important because now you can actually see any deviation from normal and prompt management will be given to the woman and thus save the foetus’ life; like for instance when the symphysis fundal height is actually growing too fast, there could be an alarm that it should be investigated further.’ (P7, FGD2)

#### Theme 2: Perceived prerequisites enabling students’ competence and confidence

The prerequisites perceived as enabling competence and confidence when performing the SFH procedure were: (1) prior theoretical knowledge of the SFH procedure; (2) correct interpretation and documentation of the SFH results; (3) an enabling therapeutic environment in which to learn the procedure; and (4) utilisation of effective clinical teaching strategies.

**Prior theoretical knowledge of the symphysis fundal height procedure:** One of the prerequisites that would enable student midwives’ competence and confidence when performing the SFH procedure was prior theoretical knowledge of the procedure. This includes detailed information received during classroom teaching on how to perform the procedure, as well as its significance. The students require adequate theoretical knowledge of the SFH procedure in the classroom, and that knowledge has to be connected to anatomy and physiology. At the same time, emphasis must be placed on the critical factors that can influence the accuracy of the fundal height measurement and interpretation of the findings. Furthermore, the importance of the positioning of the patient, the abdominal palpation and the principles of plotting the findings of the SFH measurement must be emphasised. Although some students could have enough theoretical knowledge, they expressed nervousness, which affected their confidence in performing the SFH procedure:

‘They should have that knowledge on the anatomy and physiology because some of them fail to interpret where the symphysis pubis is and to which point you measure up to. So, there should be the prerequisite knowledge before you can do SFH. And also know other factors that will affect the readings and the type of patient that we have in terms of obesity.’ (P3, FGD1)

‘They need to have enough theory to correlate the finding on the tape to the size of the foetus or the period of pregnancy, because if there is a disconnect between, you know, bringing the two together, then the measurement of the symphysis fundal height is not going to make sense.’ (P10, FGD2)

**Correct interpretation and documentation of the symphysis fundal height results:** The participants expressed the second theme as the correct interpretation and documentation of the SFH results. Students should be able to interpret the result of the SFH measurement correctly, do the recording on the clinical notes correctly and plot every measurement in the antenatal care book correctly. The participants agreed that the actual plotting, that is the placing of the measurement-indicating dots in the correct positions on the graph, was not that much of a challenge for nursing students. The correct interpretation entails the correct diagnosis of a pregnancy progressing positively in relation to the estimated date of delivery (EDD), as the height of the fundus should correspond with the gestational age if it is a normal pregnancy:

‘What I noticed, the plotting, the placing of the actual dots on the correct spots on the graph, that one was not that much of a challenge. But the interpretation, that was always an issue. Doing the procedure in terms of the manual, dexterity and the skill was okay, but when it comes to the actual interpretation of this skill, there is a lot of challenges. Competence in that area is still a challenge, which could be the reason why maybe some patients end up with the graph not plotted, because if there is no motive, usually then the action is not prioritised. So maybe if we can put more emphasis on the interpretation of the findings as lecturers, that can help, as well as our colleagues in the clinic, the registered midwives.’ (P10, FGD2)

‘We know from our practising experience that when you watch somebody doing it, you only absorb about 20%. When you listen to them, you absorb 20%. When you watch them, you absorb 20%. But if you are doing it yourself, you tend to absorb 70%. So, you will teach them how to plot after you have given them the cognitive skills. When you will give them the affective and psychomotor skills and they have to practise.’ (P5, FGD1)

‘After doing the measurement or plotting, then there must be interpretation of the findings and the relevant intervention, which she has picked up that is the problem. If there are possible problems or possible complications, should be able to interpret that accordingly.’ (P7, FGD2)

**An enabling therapeutic environment:** An enabling therapeutic environment was found to be one of the prerequisites for midwifery students’ competence and confidence when performing an SFH measurement, according to the participants. Student midwives should ensure that pregnant mothers are well positioned and calm for the examination. The environment in which the procedure is to be conducted must be conducive to the procedure, with the correct, clean equipment available. It must be a private room with the necessary equipment, and a simple tape measure is crucial for performing the SFH measurement. Warm hands, coupled with manual dexterity, are paramount:

‘Another thing that comes to mind is that students should also know about the importance of positioning the patient. The patient must be positioned in a standard way in terms of when the measurement is done, so that there are no variations that are caused by the mechanical issues. I remember I talked about the correction of dextro version that also plays an important role.’ (P9, FGD2).

‘Secondly, the requirements per equipment, like the measuring tape that needs to be available to do the SFH. I think you need to have proper resources available so that teaching can be done practically and theoretically.’ (P15, FGD3)

**Utilising the correct clinical teaching strategies:** The utilisation of the correct clinical teaching strategies enhances the ability of students to perform the SFH and interpret the results, thus building the confidence of students, as indicated by the participants from the three FGDs. The correct clinical teaching strategies include demonstration of the procedure, the use of case studies, the use of training videos, simulation of the procedure, self-directed learning and competency-based assessments coupled with direct supervision. The specific objectives and the appropriate methods of assessment are needed for the students to accomplish their nursing tasks and pass their assessments. It is necessary to photocopy the SFH chart and the maternity case record and bring them to the classroom and use them to record a demonstration of the procedure. This is useful as learning is enhanced when the students can visualise what they are being taught:

‘I think we can use visual images and videos to watch after they have been taught the knowledge and the theory and after the demonstration. They can watch a video of the SFH measurement at their own pace. Some students retain information quicker than others, they can watch the procedure repeatedly.’ (P1, FGD1)

‘As part of the Basic Antenatal Care [*BANC*] workshop, they usually show the video showing the way how SFH is done. But besides that, every student should have SFH chat so that when you give them a scenario, each one will get an opportunity to plot on the graph, and you will move around checking if they’ve entered correctly. If they still haven’t mastered it, you correct each student individually and also that promotes confidence in the student …’ (P6, FGD1)

‘They must be given a chance to practice plotting before their clinical placement.’ (P7, FGD2)

‘You can also use the case study, like the case scenarios for referencing gestational ages so that they can assess, if they can, and pick up abnormalities.’ (P11, FGD3)

‘You can also simplify it on the board by demarcating the specific areas and how to record it as per the recording chart because there is a dot, or a cross, or a circle.’ (P13, FGD3)

#### Theme 3: Perceived challenges and barriers to competence and confidence

Participants identified the perceived challenges to students’ competence and confidence in performing SFH as limited prior knowledge thus affecting confidence and competence, insufficient resources for students’ SFH training, limited SFH recording and inaccuracy by other healthcare professionals from the start and lack of standardised training in performing the SFH.

**Limited prior knowledge thus affecting confidence and competence:** Participants reported a lack of prior knowledge in performing SFH, interpretation and reporting as some students do not know the significance of plotting the charts. Symphysis fundal height graphs were not being plotted and completed after performing SFH. In some cases, registered midwives do not understand the importance of doing SFH. As a result, students are not well mentored and consequently affected in terms of competence and confidence in performing the SFH measurement:

‘You find that the patient is referred to us in the regional hospital and we are the initiator may be during the last trimester of pregnancy that is the only time that the patient’s SFH is done now thinking back what is happening in the primary health care setting. There are two questions to be asked is it the workload, is it the lack of knowledge from the registered midwives out there, who do not know the significance of doing it, is it lack in-service, there is a lot of factors, which I think should be dealt with, investigated so that they do it.’ (P5, FGD1)

**Limited resources for students’ symphysis fundal height training:** Participants highlighted the lack of resources in terms of human and equipment when it comes to training of students to be competent and gain confidence in performing SFH. There is always a shortage of skilled midwives, coupled with high patient turnover, pressure of work and overload of duties. Consequently, students were not guided enough in completing their tasks of correctly performing SFH, interpretation and recording in the maternity case record:

‘The cause can be a shortage of skilled midwives, also lack of resources available, and also lack of interest generally from the maternity side due to the pressure of work, or overload of work.’ (P15, FGD3)

**Limited symphysis fundal height recording and inaccuracy by other healthcare professionals from the start:** One of the challenges influencing students to carry out SFH competently is the lack of proper recording and inaccurate history on the mother’s maternity card. Most of the time, the ANC records were incomplete and not recorded in the maternity case record and the patients who were referred from other institutions were not monitored properly with a lot of things such as IUGR not diagnosed:

‘I think those that are not plotted probably the reason could be the fact that the health personnel who attended to the pregnant women, they do not know the significance of plotting the chart and secondly, it could be that the knowledge deficit, in as far as the plotting is concerned, that is my own view.’ (P8, FGD2)

**Attitude of the students and staff towards symphysis fundal height measurement:** Limited prior knowledge, behaviour and attitude of students make it impossible for students to perform SFH competently and with confidence:

‘The level of confidence in the assessments from my perspective is that they were nervous, they had the knowledge, but they were nervous and anxious as it was their first exposure to the maternity side being a high-risk environment.’ (P15, FGD3)

**Lack of standardised training in performing the symphysis fundal height:** Midwives are trained in different institutions as per their guidelines. Hence, there is no standardisation that applies to all institutions regarding the measurement of SFH. As a result, students receive information from the clinical staff (midwives) differently, leading to them getting confused. This consequently affects their competence and confidence to correctly perform SFH:

‘And another problem is that we trained at different institutions, so standardisation is important. Just to add, we can all know, but what we need to standardise our teaching between us and the clinical area. So, it’s important that the knowledge that we share with our students is standardised.’ (P4, FGD1)

#### Theme 4: Recommendations to improve the competence and confidence of symphysis fundal height measurement, plotting and interpretation

Key recommendations from participants to improve competence and confidence of SFH measurement, plotting and interpretation were ensuring the standardisation of practice at the operational level, availing of resources, in-service training of educators and encouraging multidisciplinary team collaborations. These recommendations also point to the gaps in the SFH measurement practice and systems, mostly at the operational level.

**Standardised practice:** Participants showed that there was a need for standardisation of practices in SFH measurement, largely at the operational level because a lot of staff are involved at various points with each visit. Participants indicated that efficient and accurate record trace and tracking was important as there were day and night shifts taking care of a woman.

Random unannounced spot checks were also proposed to ensure staff remain diligent in carrying out SFH measurement procedures. Furthermore, participants suggested that operational managers should keep oversight of the whole system by carrying out periodic audits of SFH measurement records. Another suggestion was also that SFH measurement should be considered a stand-alone procedure, so that the students can deliberate more on the SFH. In practice, SFH is not performed as a stand-alone procedure, it’s carried out in conjunction with abdominal examination, which can be complicated for the student to carry out several interpretations if not properly guided in as far as the information on abdominal examination is concerned:

‘There should be some kind of spot checks by senior staff let’s say the junior staff are recording the observations, senior staff will need to do checks.’ (P14, FGD3)

‘In the near future, we should just have that as a stand-alone procedure, so that now a student will deliberate more on the symphysis fundal height rather than focusing on other areas.’ (P8, FGD2)

**Availability of resources and in-service training of educators:** Participants indicated a need for human resource capacitation and thorough in-service training citing that interpretation of readings was more important than just doing the SFH measurement procedure. This then also meant that during the in-service trainings, staff are reminded and refreshed on how to record these observations. These trainings would be carried out for all clinical staff as well as the academic staff. It was also suggested that students be equipped with personal copies of the SFH measurement booklets because it was not easy to know where to plot, as they would often be seen flipping through pages of a document they only see for a short time. The following verbatim shows this:

‘If we can allow the students to each have their own maternity book.’ (P1, FGD1)

**Multidisciplinary team collaboration:** It was suggested that for the overall improvement of SFH measurement practice, there was a need for multidisciplinary teams’ collaboration, which helps with the quick identification of complications as well. The suggestions, while raised at Campus C, are profound in that having a multidisciplinary team collaboration enabled the medical staff to be alerted earlier of any high-risk mothers:

‘Also, okay, to also involve the medical staff, for the times they see these high-risk mothers, plot that the client has been attending to a doctor, and you find that SFH is not plotted.’ (P12, FGD3)

## Discussion

### Demographic characteristics

In total, 15 lecturers participated in the FGDs. All participants were directly involved in the theoretical or clinical teaching of the student midwives. All participants had nursing education qualification and experience in the midwifery department.

### Themes

#### Theme 1: Perceived significance of symphysis fundal height measurement, plotting and interpretation

The findings showed that the participants perceived that it is significant to measure, plot and interpret SFH because it determines if the foetus is growing well. Symphysis fundal height can be used to identify high-risk patients and be referred for further investigation for conditions such as polyhydramnios, oligohydramnios, multiple pregnancy and IUGR. This requires prompt referral and management to save the life of the foetus. Symphysis fundal height measurement is perceived as a cost-effective tool as it can be used in low-income countries where ultrasound is not available (Unger et al. 2019).

#### Theme 2: Perceived prerequisites enabling competence and confidence in performing symphysis fundal height measurement

The student midwives must know the landmarks, that is that they must measure from the highest point of the symphysis pubis to the highest point of the fundus of the uterus to obtain accurate measurements. In this study, one of the participants talked about the necessity of prerequisite knowledge of anatomy and physiology. The SANC has stipulated the number of theoretical and practical lessons that a nursing student should receive before she or he is exposed to the clinical environment. Student midwives should be able to calculate a patient’s EDD, and they require the date of the patient’s LNMP to do so. An accurate LNMP date is essential, as it is a prerequisite for calculating the gestational age of a foetus (Rada et al. 2018; Unger et al. 2019).

In this study, the participants mentioned that the theory on the SFH measurement should have been taught before the procedure was demonstrated. Folkvord and Risa (2023) confirmed that students prefer having a theoretical basis for procedures prior to performing them in clinical practice. Folkvord and Risa (2023) further reported that students gain skills through demonstration and simulation before they go into the clinical area. The findings of this study highlighted several teaching strategies, such as demonstration, simulation, videos, competency-based learning and self-directed learning, that could foster competence and confidence in student midwives when performing the SFH procedure and interpreting the results. This supported the findings of Sharma et al. (2021), Stone et al. (2020), Thrower et al. (2020) and Sharma et al. (2021), who reported that an evidence-based approach and simulation learning would help students to achieve course objectives and develop the appropriate clinical skills. Clinical outcomes or expectations in the clinical area should be clear to the students and clinical staff so that the staff can assist the students to achieve their course objectives (Mbakaya et al. 2020).

Students should accurately understand what the SFH measurement means in the context of pregnancy. It should be recorded correctly in the clinical notes in the maternity case record and also be plotted on the SFH chart at each successive visit (Cronjé, Cilliers & Du Toit 2016). A record should be made every time the SFH measurement is taken, so that the results have meaning, which is in line with the SANC regulation R4288 (Government 2005). According to the participants, plotting the results on the graph was not a major problem for the students, as most students managed to do this part correctly. The greater challenge was in understanding what the measurements meant and interpreting them in the clinical context. The interpretation includes being able to diagnose correctly whether a pregnancy is progressing normally or not. This concurred with the findings of Dlamini (2023), where there was evidence of inaccurate plotting and interpretation of the SFH results in maternity case records in that study. In this study, the participants reported that patients’ antenatal clinic records were incomplete most of the time, as the SFH results were not recorded in the maternity case records, and the patients who were referred from other institutions were not monitored properly. Similar findings were reported by Dlamini (2023).

Clinical placement assists the students to integrate their theoretical knowledge with their clinical skills and improves their midwifery practice. This can be facilitated by the availability of preceptors in the clinical settings, which is of vital importance because they supervise the students (Alkhelaiwi et al. 2024).

Preceptors are also involved in the assessments of the students, which necessitates in-service training and standardisation of their midwifery skills, including SFH measurements and reporting. Standardisation of the teaching and assessment strategies is also important because midwives are trained in different institutions, which causes confusion for the students because they receive different instructions from the midwives in the clinical settings and their lecturers. This consequently affects their competencies and confidence in their ability to perform the SFH measurements correctly. Alkhelaiwi et al. (2024) emphasised that teaching and assessment strategies and instruments should be standardised, valid and reliable.

#### Theme 3: Perceived challenges and barriers to competence

The findings of this study revealed the factors that hinder the competence of the students in SFH measurement, plotting and interpretation. They include the lack of resources, shortage of skilled midwives and the pressure of work in the maternity units, which could be the reason why the SFH charts in the maternity case records are not completed. These findings are in line with the findings of Mbakaya et al. (2020) where they mentioned the environment, equipment, standard procedures and learning tools as factors affecting student competence in Malawi and other sub-Saharan countries. However, these challenges are also experienced in high-income countries such as Norway where there is a lack of equipment and some is outdated (Mbakaya et al. 2020).

Bogren et al. (2022) also reported that implementation of the curriculum in the clinical setting was perceived as a barrier to achieving learning outcomes. Research findings demonstrate that educators often lack formal qualifications in midwifery (Sharma et al. 2021; Thrower et al. 2020). Higher education in midwifery promotes full assessment and in-depth understanding. In South East Asia, midwifery was not separated from general nursing, and hence, the midwifery qualification was not a requirement (Bogren et al. 2022). A similar problem was discovered in Ethiopia, Ghana and Malawi (Bogren et al. 2022). Assessment strategies using valid assessment methods to measure student performance in both theory and clinical learning were also identified as barriers to competence (Bogren et al. 2022). The SFH assessment should include cognitive aspect and psychomotor aspect. Findings from this research revealed that training of midwives is done in different colleges, and there is no standardisation of procedures, including SFH measurement. Other factors that were highlighted by Folkvord and Risa (2023) are that students are motivated by the good relationship and support from midwives, teachers and fellow students. This is in line with the global standards for midwifery students who need to be in a supportive learning environment with full support from the preceptors (International Confederation of Midwives 2021). In addition, Mbakaya et al. (2020) confirmed that a poor relationship between students and staff and a lack of support from clinical teachers were the barriers that prevented students from attaining their clinical outcomes.

#### Theme 4: Recommendations

There should be in-service training on SFH for the midwives and lecturers to keep them abreast with the current information and to strengthen their confidence. Clinical accompaniment of the students and continuous assessment of the SFH measurement skill should be performed according to the SANC regulations. This will assist the students to correlate theory with practice and to become competent in the interpretation of SFH. Nursing education institutions should consider assessing SFH measurement, plotting and interpretation as a stand-alone procedure to enhance students’ understanding.

### Limitations

The study was conducted only in the public nursing college of KwaZulu-Natal, and three universities in the province were excluded. The convenience sample precludes the ability to generalise findings. The FGDs were conducted on three of the 11 KZNCN campuses because data saturation was reached during the third FGD. The number of focus group members was not the same across the campuses because the sampling strategy was purposive. The sample presented some limitations as the lecturers without midwifery experience were excluded from the study.

## Conclusion

This study was conducted to explore and describe the perceptions of midwifery lecturers on the competence and confidence of student midwives in measuring, plotting and interpreting SFH results. Most lecturers perceived students to be competent at SFH measurement but lacked skill in the interpretation of results. As one participant observed, plotting measurements is a challenge, as is interpretation. Where students lack plotting and management abilities, there are potentially devastating maternal and foetal conditions that can be missed.

It is the role of midwifery faculty and lecturers to ensure that students have the requisite skills to perform and plot SFH measurements. Where abnormal measurements are observed, appropriate management is crucial to prevent potentially devastating perinatal outcomes. As part of every antenatal visit, this simple intervention has the potential to allow for early intervention.
